# Association between residual cholesterol and vulnerable non-culprit lesions progressing to major adverse cardiovascular events

**DOI:** 10.3389/fendo.2025.1603907

**Published:** 2025-08-15

**Authors:** Fengfeng Wang, Qun Li

**Affiliations:** Department of Children’s Heart Center, Fuwai Central China Cardiovascular Hospital, Zhengzhou University Central China Fuwai Hospital, Zhengzhou, China

**Keywords:** residual cholesterol, non-culprit lesion, thin-cap fibroatheroma, major adverse cardiovascular events, CVD (cardio vascular disease)

## Abstract

**Background:**

Residual cholesterol (RC), a key indicator of lipid metabolism disorders, has been increasingly implicated in atherosclerotic progression. However, its association with vulnerable thin-cap fibroatheromas (TCFA) in non-culprit coronary lesions (NCCLs) and the subsequent risk of major adverse cardiovascular events (MACE) remains insufficiently explored.

**Methods:**

In this prospective observational study conducted between June 2022 and September 2023, patients diagnosed with TCFA within NCCLs were followed for at least 12 months. Participants were grouped according to MACE occurrence. Spearman correlation and multivariate logistic regression were used to examine associations between RC levels, plaque vulnerability features, and MACE.

**Results:**

RC showed significant correlations with key vulnerability markers—negatively with fibrous cap thickness (rs = -0.61, P < 0.001) and positively with lipid arc (rs = 0.75, P < 0.001). In univariate analysis, elevated RC was associated with a 1.88-fold increased risk of MACE. RC remained an independent risk factor in multivariate analysis (OR = 1.127, 95% CI: 1.101–1.593, P = 0.031). ROC analysis yielded moderate predictive value (AUC = 0.720).

**Conclusion:**

Elevated RC is associated with greater plaque vulnerability and increased MACE risk in patients with NCCL-TCFA. These findings suggest RC’s potential role in cardiovascular risk stratification, warranting further validation in larger studies.

## 1 Introduction

Cardiovascular disease (CVD) remains a leading cause of death and disability worldwide, posing significant challenges to public health and healthcare resources due to its high prevalence and mortality rates. Despite advancements in diagnostic and therapeutic strategies, early intervention targeting the progression of CVD is critical for preventing major adverse cardiovascular events (MACE). Among patients with coronary artery disease (CAD), non-culprit lesions (NCCL) have been identified as a major source of MACE (1–3), with their rupture often leading to acute coronary syndromes (ACS) or even cardiac death (4, 5).

In the progression of NCCL, thin-cap fibroatheromas (TCFA) represent the most common type of vulnerable plaque (6). TCFA is characterized by features such as a thin fibrous cap, a large lipid core, infiltration of inflammatory cells, and the presence of calcified nodules (7). TCFAs are among the most prevalent types of vulnerable plaques observed in NCCLs (8). Studies have estimated that TCFAs may be present in up to 40% of angiographically non-obstructive NCCLs, and they are independently associated with future adverse cardiovascular outcomes even when not flow-limiting (9–11). This underscores the clinical importance of identifying high-risk features in NCCLs, which are often overlooked compared to culprit lesions.

The vulnerability of TCFA not only determines the likelihood of plaque rupture but is also closely associated with the occurrence of cardiovascular events (12–15). Therefore, identifying key factors that influence TCFA vulnerability and its progression to MACE is of paramount importance for optimizing CVD prevention strategies. It is also important to consider the current clinical context in which NCCL management varies depending on the type of acute myocardial infarction (AMI) (16–18). In STEMI patients, revascularization of severe NCCLs is typically class IA recommended, whereas in NSTEMI, the evidence is less definitive and generally regarded as class IIa (19). Furthermore, the use of fractional flow reserve (FFR) has emerged as a key tool in guiding the need for intervention, yet it primarily assesses functional ischemia and may not fully reflect the structural vulnerability of plaques such as TCFAs (17). Therefore, understanding anatomical high-risk features remains crucial in complementing physiology-based decisions.

Residual cholesterol (RC), an independent marker of metabolic dysfunction, has gained increasing attention in recent years (20–22). RC is calculated by subtracting high-density lipoprotein cholesterol (HDL-C) and low-density lipoprotein cholesterol (LDL-C) from total cholesterol (TC) and reflects the accumulation of triglyceride-rich lipoproteins, such as very-low-density lipoproteins and chylomicron remnants (23–25). Previous studies have demonstrated that elevated RC is strongly associated with the development and progression of atherosclerotic diseases (26, 27). Its potential mechanisms include inducing endothelial dysfunction, activating inflammatory responses, and promoting lipid deposition. However, systematic research investigating the relationship between RC, TCFA vulnerability in NCCL, and MACE remains lacking (1, 28–30). On the other hand, RC may drive both systemic inflammation and direct atherogenic effects, supporting its evaluation in non-culprit lesion settings (24, 31). Our study specifically focuses on this underexplored association in the context of NCCLs, rather than culprit lesions traditionally targeted during revascularization.

Therefore, this study aims to explore whether elevated RC—as a marker of remnant lipoprotein burden—contributes to structural vulnerability in NCCL-TCFAs and increases the risk of MACE. Given the increasing recognition of non-obstructive yet high-risk plaques as contributors to future events, our work seeks to fill a critical gap in understanding the metabolic and anatomical mechanisms underlying NCCL instability. The primary outcome of this study is to assess the association between RC levels and the occurrence of MACE in patients with NCCL-TCFA. The secondary outcomes include evaluating the correlation between RC and TCFA features (fibrous cap thickness, lipid arc, and plaque composition), as well as assessing RC’s potential value as a predictive biomarker for MACE risk stratification.

## 2 Methods

### 2.1 Study population

**Figure 1:**
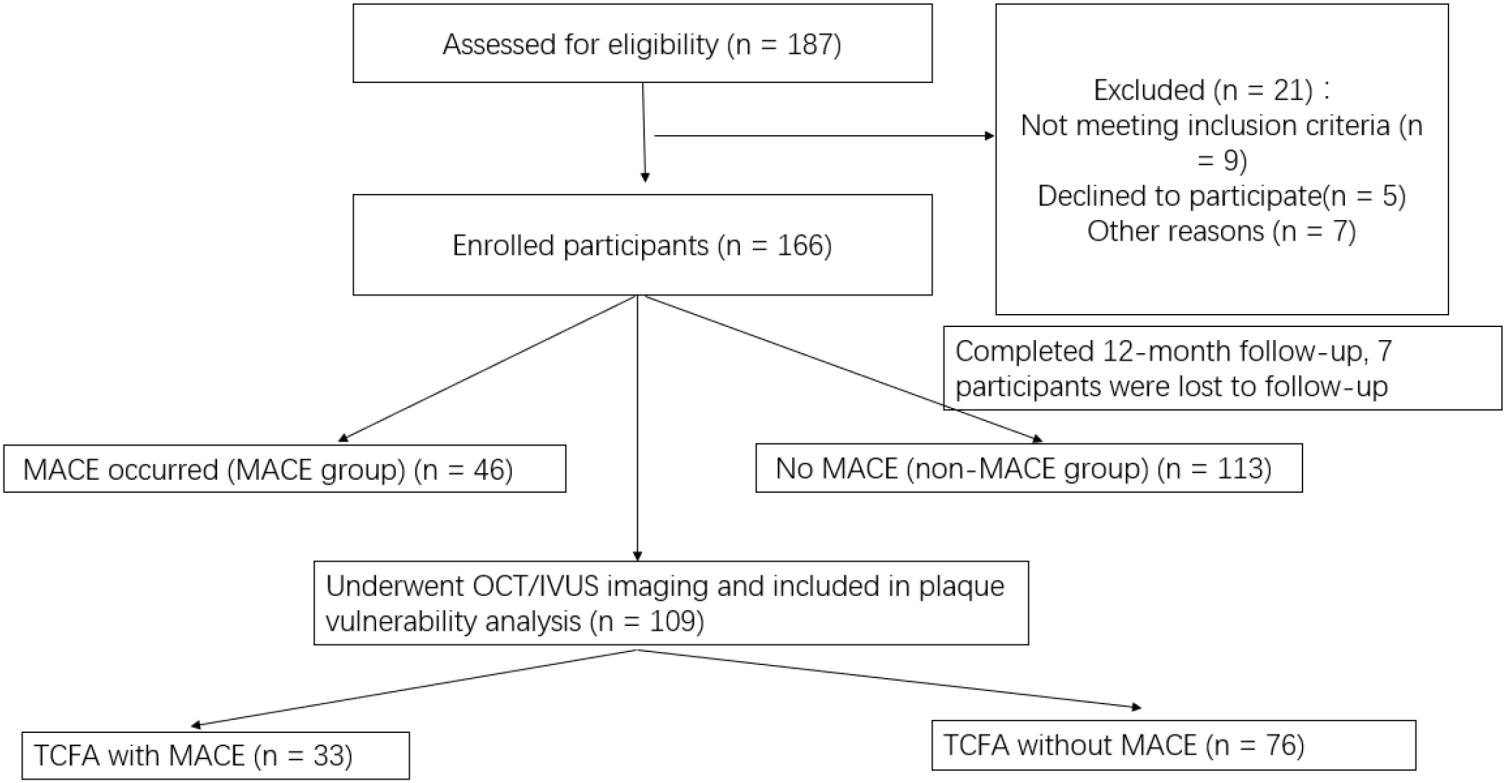
STROBE flow diagram of participant enrollment and analysis.

This was a single-center, prospective observational cohort study conducted at Fuwai Central China Cardiovascular Hospital between June 2022 and September 2023. The study protocol was approved by the Institutional Research Ethics Committee of Fuwai Central China Cardiovascular Hospital, and all procedures complied with the Declaration of Helsinki. All participants provided written informed consent prior to inclusion. Given that this was an observational study with no interventional component, no randomization was applied. Patients were enrolled consecutively based on predefined inclusion and exclusion criteria and were classified retrospectively into MACE and non-MACE groups according to whether they experienced major adverse cardiovascular events during the 12-month follow-up period. Of the 187 patients assessed for eligibility, 21 were excluded based on predefined criteria. An additional 7 participants were lost to follow-up due to incomplete imaging or laboratory data, resulting in 159 patients included in the final analysis. In total, 159 patients were included in the final analysis, of whom 46 patients experienced MACE (MACE group) and 113 patients did not (non-MACE group) (See **Figure 1**).

### 2.2 Inclusion criteria

All eligible patients were enrolled consecutively during the study period to reduce selection bias. The inclusion criteria were as follows: (1) confirmation of non-culprit lesions (NCCL) by coronary angiography, defined as coronary lesions not causing the patient’s current symptoms; (2) identification of TCFA within NCCL through imaging examinations, such as intravascular ultrasound (IVUS) or optical coherence tomography (OCT); (3) completion of imaging evaluations and blood biochemical tests during follow-up; (4) a follow-up period of no less than 12 months; and (5) provision of written informed consent and voluntary participation in the study.

### 2.3 Exclusion criteria

The exclusion criteria included any of the following: (1) patients with acute coronary syndromes (ACS) who had not received stabilized treatment; (2) those with severe cardiac, pulmonary, hepatic, or renal dysfunction or other serious systemic diseases; (3) patients with malignant tumors or confirmed infectious diseases; (4) those unable to complete imaging examinations or blood testing; and (5) patients with poor adherence to the study or those lost to follow-up.

### 2.4 Patient grouping

Patients were divided into two groups based on whether they experienced major adverse cardiovascular events (MACE) during the follow-up period. The MACE group included patients with MACE events attributed to NCCL, such as cardiac death, nonfatal myocardial infarction, and unplanned coronary revascularization. The non-MACE group consisted of patients who did not experience these events during the follow-up period. The MACE group included patients who experienced MACE, defined as cardiac death, nonfatal myocardial infarction, or unplanned coronary revascularization, during follow-up.

### 2.5 Data collection

#### 2.5.1 Baseline characteristics

Clinical baseline characteristics of all patients were collected, including sex, age, body mass index (BMI), smoking status, alcohol consumption, diabetes, hypertension, dyslipidemia, and family history of cardiovascular disease. These data were obtained from the patients’ electronic medical records and hospitalization records. To ensure accuracy, the baseline characteristics were independently verified and cross-checked by two researchers.

Medication use at baseline was recorded, including statins, dual antiplatelet therapy (DAPT), beta-blockers, and ACE inhibitors/ARBs. In our cohort, 82.4% of patients were on statins, and 91.2% received antiplatelet therapy during hospitalization.

#### 2.5.2 Biochemical parameters

Venous blood samples were collected from patients upon admission to measure biochemical parameters, including: residual cholesterol (RC), total cholesterol (TC), triglycerides (TG), high-density lipoprotein cholesterol (HDL-C), low-density lipoprotein cholesterol (LDL-C), blood glucose, and glycated hemoglobin (HbA1c). RC was calculated as TC minus HDL-C and LDL-C. TC and TG were measured using enzymatic methods, HDL-C and LDL-C were measured using direct assays, while blood glucose and HbA1c were determined using the glucose oxidase method and high-performance liquid chromatography, respectively. All blood tests were performed after an 8-hour overnight fast and analyzed at a hospital-certified central laboratory. Each sample was tested in duplicate to ensure accuracy, with the average value used for analysis.

#### 2.5.3 Imaging parameters

All patients underwent coronary angiography and plaque imaging assessments using optical coherence tomography (OCT) or intravascular ultrasound (IVUS). Plaque characteristics analyzed included the thinnest fibrous cap thickness, measured by OCT to evaluate the risk of plaque rupture, and the maximum lipid arc, representing the largest angle of the lipid core in cross-sectional views. Lumen stenosis rate was calculated as the ratio of the minimal lumen area (MLA) at the lesion site to the normal reference lumen area. Additional parameters such as calcified nodules, macrophage infiltration, cholesterol crystals, and microvessel density were identified or qualitatively assessed based on imaging data. Imaging analyses were independently conducted by two experienced radiologists, and any discrepancies were resolved by a third radiologist. Among the enrolled patients, 89 underwent optical coherence tomography (OCT) and 70 underwent intravascular ultrasound (IVUS). In cases where both modalities were available, OCT data were prioritized for fibrous cap thickness and lipid arc evaluation due to its superior spatial resolution.

In our study, TCFA was defined based on established OCT and IVUS criteria. For OCT, TCFA was characterized by a lipid-rich plaque with a lipid arc >180° and a fibrous cap thickness <65 μm. For IVUS-based assessments, virtual histology-defined TCFA was identified by a necrotic core in contact with the lumen in the presence of thin fibrous cap, as per standard definitions.

#### 2.5.4 Data quality control

To ensure the quality of collected data, several measures were implemented. First, all data were double-entered by independent research assistants and subjected to cross-checking for consistency. Regular multicenter meetings were held to standardize data collection and processing procedures, ensuring uniformity across study sites. Additionally, a dedicated data management system was established to enable real-time monitoring, review, and quality assessment of key data points.

### 2.6 Statistical analysis

All statistical analyses were performed using SPSS version 26.0 (IBM Corp., Armonk, NY) and R version 4.2.1. Normality of continuous variables was assessed using the Shapiro–Wilk test. Data with a normal distribution were presented as mean ± standard deviation (SD) and compared using the independent samples t-test. Non-normally distributed variables were reported as median with interquartile ranges (IQR) and analyzed using the Mann–Whitney U test. Categorical variables were expressed as frequencies (percentages) and compared using the chi-square test or Fisher’s exact test, depending on the expected counts. Correlations between RC and vulnerable plaque features (e.g., fibrous cap thickness, lipid arc) were assessed using Spearman’s rank correlation coefficient (rs). To evaluate the independent association between RC and MACE, multivariate logistic regression was conducted, adjusting for potential confounders (e.g., age, hypertension, diabetes). Results were reported as odds ratios (OR) with corresponding 95% confidence intervals (CI). Predictive performance of RC was assessed using receiver operating characteristic (ROC) curve analysis, with the area under the curve (AUC) interpreted as follows: AUC > 0.7 indicates moderate prediction, and AUC > 0.8 indicates good prediction ability. All statistical tests were two-tailed, and P-values < 0.05 were considered statistically significant.

The primary hypothesis was that RC is independently associated with both greater TCFA plaque vulnerability and increased risk of MACE in patients with non-culprit coronary lesions. Based on previous literature and assuming an expected effect size (OR) of 1.9, a statistical power of 80% (β = 0.20), and a two-sided α level of 0.05, the minimum sample size required for logistic regression analysis was calculated using the formula for proportions in two independent groups. This yielded a minimum required sample size of 136 patients. Considering a 15% loss to follow-up rate, we aimed to recruit at least 156 participants, ultimately including 159.

To address potential confounding, variables known to influence MACE risk—including age, sex, diabetes, hypertension, lipid parameters, and smoking history—were included in the multivariate logistic regression model. Variable selection was based on clinical relevance and univariate screening (P < 0.10). To evaluate multicollinearity among independent variables, Variance Inflation Factor (VIF) was calculated. All variables included in the final model had VIF values less than 2, indicating an acceptable level of collinearity.

## 3 Results

### 3.1 Baseline characteristics comparison between MACE and non-MACE groups

In the comparison of baseline data between patients with MACE (N=46) and those without MACE (N=113), no significant differences were observed in age (55.39 ± 10.45 years *vs*. 56.91 ± 9.23 years, p=0.697), BMI (24.56 ± 4.29 kg/m² *vs*. 24.89 ± 4.10 kg/m², p=0.361), TG (2.15 ± 1.65 mmol/L *vs*. 1.89 ± 1.16 mmol/L, p=0.536), HDL-C (0.92 ± 0.29 mmol/L *vs*. 0.92 ± 0.23 mmol/L, p=0.509), or HbA1c (1.90% *vs*. 2.40%, p=0.128). However, the MACE group exhibited significantly higher levels of TC (3.9 ± 1.77 mmol/L *vs*. 3.53 ± 0.99 mmol/L, p=0.001), LDL-C (2.74 ± 1.52 mmol/L *vs*. 2.02 ± 0.82 mmol/L, p=0.007), RC (0.6 ± 0.48 mmol/L *vs*. 0.56 ± 0.21 mmol/L, p=0.001), and TCFA (6.66 ± 1.68% *vs*. 5.82 ± 1.14%, p=0.019) compared to the non-MACE group. Additionally, there were no significant differences in baseline medication use between groups, with statin use observed in 37 (80.4%) of MACE patients versus 94 (83.2%) of non-MACE patients (P = 0.641), and antiplatelet use in 41 (89.1%) versus 104 (92.0%) respectively (P = 0.507) (**Table 1**).

**Table 1:** Comparison of baseline data between patients with MACE and those without MACE.

Variable	MACE group N=46	Non-MACE group N-113	P-value
Age (years)	55.39 ± 10.45	56.91 ± 9.23	0.697
BMI (kg/m²)	24.56 ± 4.29	24.89 ± 4.10	0.361
TG (mmol/L)	2.15 ± 1.65	1.89 ± 1.16	0.536
TC (mmol/L)	3.9 ± 1.77	3.53 ± 0.99	0.001
HDL-C (mmol/L)	0.92 ± 0.29	0.92 ± 0.23	0.509
LDL-C (mmol/L)	2.74 ± 1.52	2.02 ± 0.82	0.007
RC (mmol/L)	0.6 ± 0.48	0.56 ± 0.21	0.001
HbA1c (%)	1.90 (1.40, 6.10)	2.40 (0.60, 6.50)	0.128
TCFA (%)	6.66 ± 1.68	5.82 ± 1.14	0.019
Statin use	37 (80.4%)	94 (83.2%)	0.641
Antiplatelet use	41 (89.1%)	104 (92.0%)	0.507

BMI, body mass index; TG, triglycerides; TC, total cholesterol; HDL-C, high-density lipoprotein cholesterol; LDL-C, low-density lipoprotein cholesterol; RC, residual cholesterol; HbA1c, glycated hemoglobin; TCFA, thin-cap fibroatheroma.

Continuous variables were compared using independent samples t-test or Mann–Whitney U test depending on distribution; categorical variables were compared using χ² test or Fisher’s exact test. P < 0.05 considered statistically significant

### 3.2 The baseline data of the TCFA group with MACE and the TCFA group without MACE variable

A total of 109 patients with confirmed TCFA and complete high-quality OCT/IVUS imaging were included in a subgroup analysis of plaque vulnerability characteristics (**Table 2**), derived from the overall study cohort of 159 participants. In the comparison of baseline data between the TCFA with MACE group (n=33) and the TCFA without MACE group (n=76), no significant differences were observed in age (54.90 ± 10.70 years *vs*. 55.20 ± 9.50 years, p=0.068), family history (39.1% *vs*. 43.4%, p=0.143), smoking history (50.0% *vs*. 48.2%, p=0.9), hypertension (63.6% *vs*. 61.4%, p=0.746), hyperlipidemia (54.5% *vs*. 69.9%, p=0.185), TG (1.85 ± 0.73 mmol/L *vs*. 1.72 ± 0.81 mmol/L, p=0.66), HDL-C (0.95 ± 0.20 mmol/L *vs*. 0.99 ± 0.24 mmol/L, p=0.601), LDL-C (2.55 ± 1.10 mmol/L *vs*. 2.25 ± 0.80 mmol/L, p=0.085), HbA1c (6.42 ± 1.33% *vs*. 5.90 ± 1.00%, p=0.146), thinnest fibrous cap thickness (49.00 ± 3.80 μm *vs*. 52.20 ± 3.10 μm, p=0.095), maximum lipid arc (195.50 ± 18.00° *vs*. 191.00 ± 20.50°, p=0.295), lumen stenosis rate (56.50 ± 8.00% *vs*. 54.90 ± 8.60%, p=0.421), MLA (3.70 ± 0.75 mm² *vs*. 3.95 ± 1.10 mm², p=0.273), calcified nodules (54.5% *vs*. 41.0%, p=0.234), macrophage infiltration (77.3% *vs*. 68.7%, p=0.444), microvessels (59.1% *vs*. 43.4%, p=0.225), or cholesterol crystals (31.8% *vs*. 33.7%, p=0.839). However, the TCFA with MACE group had a significantly higher prevalence of diabetes (54.5% *vs*. 30.1%, p=0.038), as well as higher levels of TC (3.97 ± 1.20 mmol/L *vs*. 3.51 ± 0.90 mmol/L, p=0.023) and RC (0.72 ± 0.35 mmol/L *vs*. 0.50 ± 0.27 mmol/L, p=0.021) compared to the TCFA without MACE group. (**Table 2**).

**Table 2:** The baseline data of the TCFA group with MACE and the TCFA group without MACE.

Variable	TCFA with MACE (n=33)	TCFA without MACE (n=76)	P-value
Age (years)	54.90 ± 10.70	55.20 ± 9.50	0.068
Family History (%)	13 (39.1%)	36 (43.4%)	0.143
Smoking History (%)	11 (50.0%)	40 (48.2%)	0.9
Hypertension (%)	14 (63.6%)	51 (61.4%)	0.746
Diabetes (%)	12 (54.5%)	25 (30.1%)	0.038
Hyperlipidemia (%)	12 (54.5%)	58 (69.9%)	0.185
TG (mmol/L)	1.85 ± 0.73	1.72 ± 0.81	0.66
TC (mmol/L)	3.97 ± 1.20	3.51 ± 0.90	0.023
HDL-C (mmol/L)	0.95 ± 0.20	0.99 ± 0.24	0.601
LDL-C (mmol/L)	2.55 ± 1.10	2.25 ± 0.80	0.085
RC (mmol/L)	0.72 ± 0.35	0.50 ± 0.27	0.021
HbA1c (%)	6.42 ± 1.33	5.90 ± 1.00	0.146
Thinnest Fibrous Cap Thickness (μm)	49.00 ± 3.80	52.20 ± 3.10	0.095
Maximum Lipid Arc (°)	195.50 ± 18.00	191.00 ± 20.50	0.295
Lumen Stenosis Rate (%)	56.50 ± 8.00	54.90 ± 8.60	0.421
MLA (mm²)	3.70 ± 0.75	3.95 ± 1.10	0.273
Calcified Nodules (%)	12 (54.5%)	34 (41.0%)	0.234
Macrophage Infiltration (%)	17 (77.3%)	57 (68.7%)	0.444
Microvessels (%)	13 (59.1%)	36 (43.4%)	0.225
Cholesterol Crystals (%)	7 (31.8%)	28 (33.7%)	0.839

BMI, body mass index; TG, triglycerides; TC, total cholesterol; HDL-C, high-density lipoprotein cholesterol; LDL-C, low-density lipoprotein cholesterol; RC, residual cholesterol; HbA1c, glycated hemoglobin; TCFA, thin-cap fibroatheroma.

Continuous variables were compared using independent samples t-test or Mann–Whitney U test depending on distribution; categorical variables were compared using χ² test or Fisher’s exact test. P < 0.05 considered statistically significant

### 3.3 Association between residual cholesterol and TCFA characteristics

In the correlation analysis of TCFA plaque characteristics with RC and NCCLs, the results showed that the thinnest fibrous cap thickness was significantly negatively correlated with RC (r = -0.610, p < 0.001), while the maximum lipid arc was significantly positively correlated with RC (r = 0.747, p < 0.001). The lumen stenosis rate showed a positive trend but did not reach statistical significance (r = 0.510, p = 0.059), and MLA was significantly negatively correlated with RC (r = -0.767, p < 0.001). Additionally, macrophage infiltration was significantly positively correlated with RC (r = 0.413, p < 0.001), whereas calcified nodules (r = 0.159, p = 0.105), microvessels (r = 0.198, p = 0.098), and cholesterol crystals (r = 0.143, p = 0.285) did not show statistically significant correlations with RC (**Table 3**).

**Table 3:** Correlation analysis results of TCFA plaque characteristics in RC and NCCLs.

Variable	r value	P
Thinnest Fibrous Cap	-0.610	<0.001
Maximum Lipid Arc	0.747	<0.001
Lumen Stenosis Rate	0.510	0.059
MLA	-0.767	<0.001
Calcified Nodules	0.159	0.105
Macrophage Infiltration	0.413	<0.001
Micro-vessels	0.198	0.098
Cholesterol Crystals	0.143	0.285

Spearman’s rank correlation coefficient (rs) was used for non-parametric correlation analysis. P < 0.05 was considered statistically significant.”

### 3.4 Multivariate logistic regression for MACE in non-culprit lesion TCFA

In the multivariate logistic regression analysis for the occurrence of MACE in TCFA within NCCLs, univariate analysis showed that hypertension (OR = 1.951, 95%CI: 1.410 ~ 4.140, p = 0.008) and RC (remnant cholesterol, OR = 1.880, 95%CI: 1.440 ~ 1.070, p = 0.019) were significantly associated with the occurrence of MACE. However, age, sex, family history, smoking history, diabetes, TG, TC, HDL-C, and LDL-C did not show significant correlations (p > 0.05). In the multivariate logistic regression analysis, RC remained an independent predictor of MACE (OR = 1.127, 95%CI: 1.101 ~ 1.593, p = 0.031), while the significance of hypertension was lost (OR = 1.671, 95%CI: 0.056 ~ 1.418, p = 0.231) (**Table 4**).

**Table 4:** Multivariate logistic regression analysis for TCFA occurrence of MACE in NCCLs.

Variable	Univariate logistic regression		Multivariate logistic regression	
	OR (95%CI)	P	OR (95%CI)	P
Age	1.220 (0.270 ~ 1.600)	0.826		
Sex	1.400 (0.370 ~ 1.340)	0.609		
Family History	2.220 (0.720 ~ 3.870)	0.784		
Smoking History	0.981(0.260 ~ 2.620)	0.830		
Hypertension	1.951(1.410 ~ 4.140)	0.008	1.671 (0.056 ~1.418)	0.231
Diabetes	1.410 (0.170 ~ 2.990)	0.832		
TG	1.150 (0.670 ~ 2.000)	0.696		
TC	1.470 (0.970 ~ 2.250)	0.089		
HDL-C	0.710 (0.120 ~ 4.210)	0.745		
LDL-C	1.430 (0.880 ~ 2.320)	0.158		
RC	1.880 (1.440 ~ 1.070)	0.019	1.127 (1.101 ~ 1.593)	0.031

RC, Residual Cholesterol; MACE, Major Adverse Cardiovascular Events.

Logistic regression analysis performed. P < 0.05 considered significant. Multicollinearity was assessed (VIF < 2).

### 3.5 Predictive value of residual cholesterol for MACE in TCFA: ROC curve analysis

**Figure 2:**
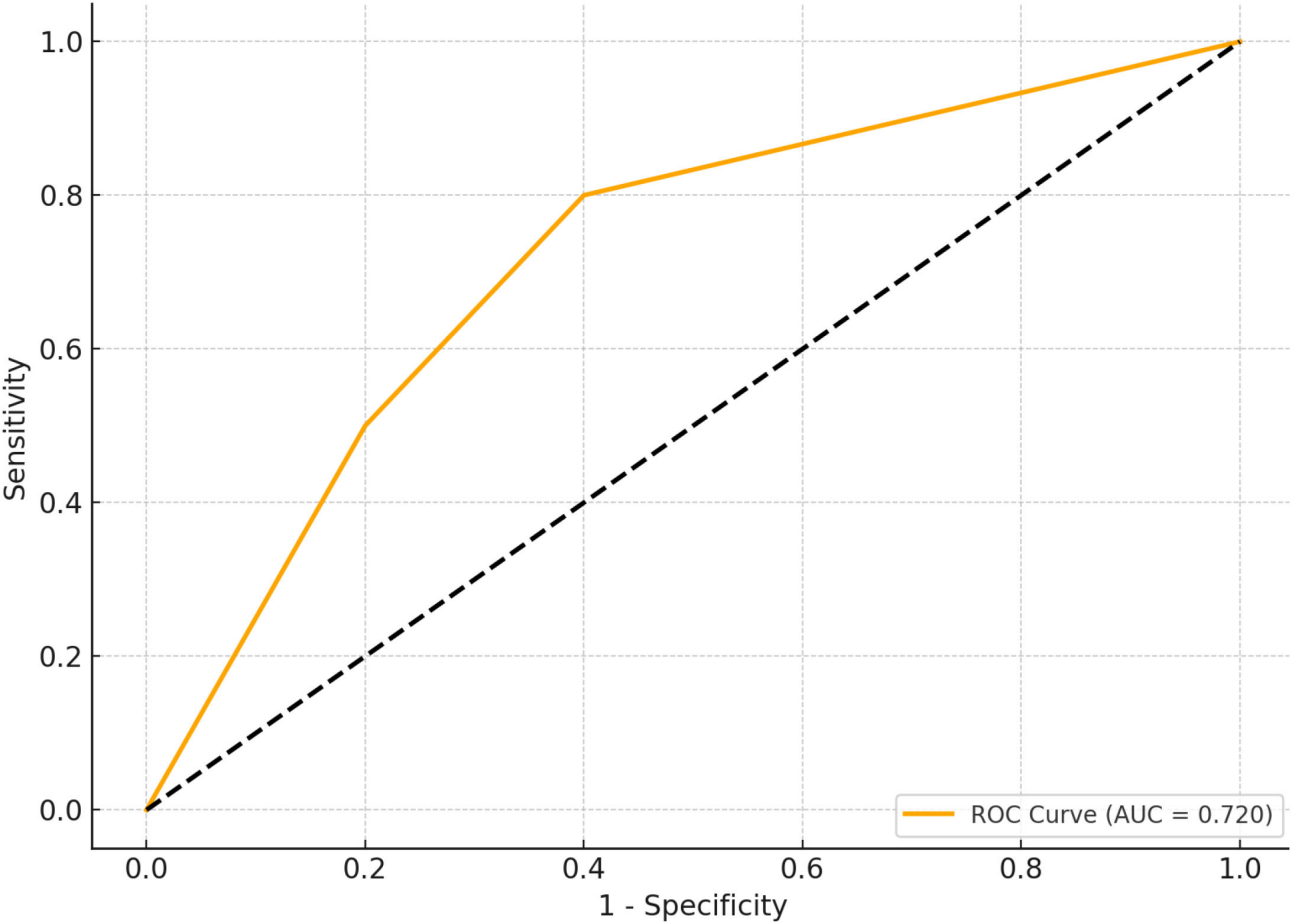
ROC curve simulation for RC predictive value in MACE. **Figure 1** presents the simulated receiver operating characteristic (ROC) curve for residual cholesterol (RC) in predicting the occurrence of major adverse cardiovascular events (MACE) within thin-cap fibroatheromas (TCFA) of non-culprit lesions (NCCLs). ROC analysis conducted to assess predictive performance. Significance determined at P < 0.05.

**Figure 1** depicts the receiver operating characteristic (ROC) curve analysis evaluating the predictive value of RC for MACE occurring in TCFA of NCCLs. The ROC curve demonstrated moderate predictive performance, with an area under the curve (AUC) of 0.720, indicating RC as a potential biomarker for risk stratification and prognosis of MACE in patients with TCFA in NCCLs (**Figure 2**; **Table 5**).

**Table 5:** ROC curve analysis for residual cholesterol in predicting MACE.

Parameter	Value
AUC	0.720
95% CI	0.631–0.809
P-value	< 0.001
Optimal Cut-off (mmol/L)	0.68
Sensitivity	69.7%
Specificity	72.4%

AUC, Area Under the Curve; CI, Confidence Interval; RC, Residual Cholesterol; MACE, Major Adverse Cardiovascular Events.

## 4 Discussion

In this study, we observed that elevated RC was significantly associated with features of plaque vulnerability, including thinner fibrous caps and larger lipid arcs in TCFAs of NCCLs. Moreover, RC emerged as an independent risk factor for MACE, with a modest predictive ability demonstrated by ROC analysis (AUC = 0.720). These findings underscore the potential role of RC in identifying high-risk NCCLs and guiding secondary prevention strategies. This study explored the relationship between RC and the vulnerability of TCFAs in NCCLs, as well as its association with the risk of MACE, offering new perspectives for targeted intervention in vulnerable NCCL plaques. Our findings clarify, for the first time, the impact of RC on critical features of TCFA vulnerability—specifically, fibrous cap thickness and lipid arc—and identify RC as an independent risk factor for MACE. These results suggest that RC may serve not only as a predictive marker for NCCL vulnerability and cardiovascular event risk, but also as a strategic target for refining CVD prevention and intervention. Furthermore, by integrating imaging data with biochemical indices, this study reveals potential mechanisms of RC in the progression of vulnerable NCCLs, providing fresh evidence for investigating the role of RC in atherosclerotic diseases.

Although RC was treated as a continuous variable in this study, our results raise the question of whether a threshold effect may exist—i.e., a critical level of RC above which the risk of MACE increases disproportionately (32, 33). Prior studies suggest that RC > 0.8 mmol/L may confer elevated risk even in patients with well-controlled LDL-C, but further work is needed to define optimal cut-off values across diverse populations. Compared with other emerging biomarkers of residual cardiovascular risk such as lipoprotein(a) [Lp(a)] and apolipoprotein B (apoB), RC has the advantage of being routinely available from standard lipid panels and easy to calculate (34, 35). While Lp(a) and apoB have been associated with MACE in various cohorts, RC reflects the cholesterol content of remnant lipoproteins and may uniquely capture postprandial and triglyceride-rich atherogenic burden (36, 37). Future studies comparing the relative predictive value and combinatory models of these markers are warranted.

### 4.1 Influence of residual cholesterol on TCFA vulnerability

RC, an important indicator of lipid metabolism, is closely associated with the onset and progression of atherosclerosi (38)s. In this study, we found that RC levels significantly affect the key vulnerability features of TCFAs. Elevated RC may exacerbate TFCA instability through the following mechanisms:

First, RC is primarily composed of triglyceride-rich lipoprotein particles (e.g., very-low-density lipoproteins and chylomicron remnants). Metabolic abnormalities can lead to the accumulation of these remnants in the bloodstream (7, 8). Given their high arterial wall permeability, these particles can easily penetrate the endothelium and undergo oxidation, inducing local inflammatory responses and macrophage activation, thereby expanding the lipid core and thinning the fibrous cap (10, 11). Additionally, RC may promote further instability of the fibrous cap by increasing endothelial cell permeability and enhancing smooth muscle cell chemotaxis (9).

In this study, RC levels were significantly and negatively correlated with the thinnest fibrous cap thickness, suggesting that elevated RC may directly compromise plaque integrity by accelerating collagen degradation and inhibiting fibrous cap formation, ultimately increasing the risk of plaque rupture (39). Simultaneously, the significant positive correlation between RC levels and the maximum lipid arc implies that higher RC may drive lipid core enlargement, further raising the probability of plaque rupture and potentially exacerbating local inflammation in a self-reinforcing cycle (40, 41). Therefore, the pivotal role of RC in lipid metabolism and atherosclerotic progression indicates that its impact on TCFA vulnerability chiefly involves thinning of the fibrous cap and expansion of the lipid core. These findings provide new evidence for clarifying the mechanisms by which RC affects coronary artery lesions and suggest that RC may serve as a potential biomarker for evaluating plaque vulnerability.

### 4.2 Residual cholesterol and MACE risk

Our study indicates that RC significantly influences the incidence of MACE, with elevated RC serving as an independent risk factor for the progression of TCFAs in NCCLs to MACE (42, 43). RC holds notable potential value in NCCL risk management, especially regarding its synergistic effect with diabetes on the progression of vulnerable plaques.

RC and diabetes may jointly accelerate the formation and deterioration of vulnerable plaques. Patients with diabetes often exhibit insulin resistance and metabolic disorders, further elevating serum RC and the accumulation of triglyceride-rich particles (44, 45). Previous research suggests that the pro-inflammatory and pro-oxidative effects of RC in diabetic patients are more pronounced, as evidenced by increased lipid core expansion, persistent inflammatory cell infiltration, and a more rapid thinning of the fibrous cap. This synergistic process may explain why both diabetes and RC emerged as independent risk factors for MACE in our study (46–48). Elevated RC not only correlates closely with TCFA vulnerability, but may also increase MACE risk through direct effects on hemodynamics and the coronary microcirculation (49). The accumulation of RC can lead to endothelial dysfunction, endothelial cell apoptosis, and local inflammatory spread, further aggravating hemodynamic abnormalities in the affected coronary regions (50, 51). These mechanisms likely play a pivotal role in driving NCCL plaque progression toward MACE.

Given the clinical relevance of RC, this study suggests that RC could serve as a potential target in NCCL risk management. On the one hand, dynamic monitoring of RC levels may facilitate the early identification of high-risk patients (52, 53). On the other hand, interventions aimed at RC—such as optimizing lipid-lowering therapies or modulating lipid metabolic pathways—could offer effective strategies for the precise prevention of plaque vulnerability and MACE risk (54). Moreover, incorporating RC into cardiovascular risk assessment models could enhance the identification of high-risk individuals, thereby aiding in the refinement of preventative strategies against cardiovascular events. Therefore, the strong association between RC and MACE indicates that RC is not only a critical marker of NCCL risk but also a promising target for precision interventions in cardiovascular disease, offering a new avenue for optimizing vulnerability management in NCCLs (55–60). However, given the modest AUC and the relatively small sample size, these findings should be interpreted cautiously. The predictive performance of RC requires further validation in larger prospective cohorts with sufficient power.

### 4.3 Residual cholesterol, pro-inflammatory pathways, and immune modulation

Emerging evidence suggests that the impact of RC on atherosclerotic plaque vulnerability may extend beyond lipid accumulation and include inflammatory and immune-modulatory mechanisms (61). Notably, RC is known to induce endothelial dysfunction, partly by stimulating the expression of vascular endothelial growth factor (VEGF), a potent pro-inflammatory mediator that promotes vascular permeability and neovascularization within plaques (62). This process may enhance fibrous cap thinning and increase microvessel density, contributing to plaque instability (63, 64). Given that low-density lipoprotein (LDL) particles—particularly oxidized LDL—act as immunogenic triggers, the atherosclerotic plaque may in part represent a chronic immune response to lipid antigens (65, 66). These findings suggest that RC-mediated pathways intersect with vascular inflammation and immune modulation, underscoring the complex interplay between metabolic and immunological drivers of plaque vulnerability.

### 4.4 Study limitations

Despite providing novel insights, this study has several limitations that must be acknowledged: First, the single-center design and relatively small sample size may limit the generalizability and statistical power, especially for subgroup analyses and ROC evaluation. Larger multicenter cohorts are needed to validate our findings. Second, although our multivariate regression model adjusted for key clinical variables, the observational and non-randomized nature of the study means residual confounding cannot be fully excluded. Important unmeasured factors such as inflammatory markers, medication adherence, or dietary patterns may still influence outcomes. Third, no external validation cohort was used to confirm the predictive performance of RC for MACE, which should be addressed in future research. Fourth, although we assessed multicollinearity using VIF and found no significant collinearity, the relatively small event number may increase the risk of model overfitting. Finally, imaging assessments (OCT and IVUS) are subject to operator dependency and interpretation bias, even though image analysis was independently conducted by two experienced radiologists. Imaging assessments predominantly relied on OCT and IVUS, which, despite their high accuracy in quantifying plaque characteristics, may carry a degree of subjectivity. More advanced imaging modalities or artificial intelligence–aided analysis could be employed in future studies to minimize potential biases.

## 5 Conclusion

In conclusion, in this prospective observational study, elevated RC levels were associated with increased vulnerability of TCFAs and a higher incidence of MACE in non-culprit coronary lesions. These findings suggest that RC may serve as a potential biomarker for risk stratification, particularly in identifying patients with metabolically active, high-risk plaques. However, due to the non-randomized and observational nature of the study, these associations should be interpreted with caution, and no causal inferences can be drawn. Further prospective, multicenter studies with larger cohorts and mechanistic validation are warranted to confirm the predictive utility and clinical significance of RC in cardiovascular risk assessment.

## Data Availability

The original contributions presented in the study are included in the article/supplementary material. Further inquiries can be directed to the corresponding author.
